# Endothelial-Mesenchymal Transition in Regenerative Medicine

**DOI:** 10.1155/2016/6962801

**Published:** 2016-04-07

**Authors:** Damian Medici

**Affiliations:** ^1^Department of Orthopaedics, The Warren Alpert Medical School of Brown University, Providence, RI 02903, USA; ^2^Division of Hematology/Oncology, Department of Medicine, The Warren Alpert Medical School of Brown University, Providence, RI 02903, USA; ^3^Center for Regenerative Medicine, The Warren Alpert Medical School of Brown University, Providence, RI 02903, USA; ^4^Cardiovascular Research Center, The Warren Alpert Medical School of Brown University, Providence, RI 02903, USA

## Abstract

Endothelial-mesenchymal transition (EndMT) is a fundamental cellular mechanism that regulates embryonic development and diseases such as cancer and fibrosis. Recent developments in biomedical research have shown remarkable potential to harness the EndMT process for tissue engineering and regeneration. As an alternative to traditional or artificial stem cell therapies, EndMT may represent a safe method for engineering new tissues to treat degenerative diseases by mimicking a process that occurs in nature. This review discusses the signaling mechanisms and therapeutic inhibitors of EndMT, as well as the role of EndMT in development, disease, acquiring stem cell properties and generating connective tissues, and its potential as a novel mechanism for tissue regeneration.

## 1. Introduction

Endothelial cells line the interior of blood vessels and lymphatic vessels [1]. Endothelial cell plasticity plays a critical role in various developmental and pathological processes [2]. EndMT is defined by the loss of cellular adhesion and cytoskeletal reorganization of actin and intermediate filaments that convert apical-basal polarity to front end-back end polarity to form spindle-shaped cells. During this transformation, there is a marked decrease in endothelial biomarkers such as VE-cadherin, CD31, TIE1, and vWF, as well as increased expression of mesenchymal biomarkers such as CD44, vimentin, FSP1, and *α*-SMA [3]. The basal lamina, primarily composed of type IV collagen and laminin, is cleaved by secreted matrix metalloproteinases (MMPs) and replaced by extracellular matrix composed of type I and type III collagen and fibronectin, which promotes cell motility [4]. These cells also acquire stem cell properties by expressing mesenchymal stem cell biomarkers and gaining multipotency [5]. This transformation is reversible through a process known as mesenchymal-endothelial transition, which is an important mechanism that regulates cardiac neovascularization [6].

*Signaling Mechanisms of EndMT*. A number of autocrine or paracrine signaling molecules can induce EndMT. These may be produced by tissue injury or immune cells recruited to the sight of injury in response to inflammation [7]. The most common cytokines that stimulate EndMT are the Transforming Growth Factor-Beta (TGF-*β*) superfamily of proteins, which include isoforms TGF-*β*1 and TGF-*β*2 as well as Bone Morphogenetic Proteins (BMPs) BMP2, BMP4, BMP6, BMP9, and BMP10 [8–14]. Other signaling pathways such as Wnt/*β*-catenin [15], Notch [16], and various receptor tyrosine kinases [17] have also been shown to activate EndMT. All of these pathways induce expression of transcription factors such as Snail, Slug, Twist, LEF-1, ZEB1, and ZEB2 that cause the repression of endothelial genes and/or expression of mesenchymal genes [17, 18]. These identified pathways allow for therapeutic targeting with the potential to inhibit this process for the treatment of EndMT-related pathologies.

Several microRNAs have been described to regulate endothelial plasticity. miR-9, a microRNA regulated by Tumor Necrosis Factor-*α* (TNF-*α*) signaling, induces EndMT in lymphatic endothelial cells [19]. miR-21 targets PTEN and mediates EndMT induced by TGF-*β* signaling [20]. miR-31 targets VAV3 to control actin remodeling and promotes the secretion of various inflammatory cytokines that promote EndMT [21].

Other positive regulators of EndMT include bleomycin, which promotes EndMT through activation of the mTOR signaling pathway [22]. Safrole oxide induces EndMT by initiating the ATF4/p75NTR/IL-8 pathway [23]. Parathyroid hormone (PTH) stimulates EndMT by enhancing nuclear localization of *β*-catenin [24]. The Kaposi sarcoma herpesvirus has been shown to induce EndMT by enhancing Notch signaling [16].

Physiological processes such as endothelial cell apoptosis can also cause EndMT through the upregulation of TGF-*β*1 in both apoptotic cells and in the adjacent viable cells [25]. Fluid shear stress studies have shown no EndMT with laminar fluid shear stress but induction of EndMT with disturbed flow shear stress [26]. Ventricular mechanical stretching causes EndMT associated with dyssynchronous heart failure [27]. High glucose levels can cause endothelial cell damage and subsequent stimulation of EndMT [28]. Hypoxia associated with tissue damage, ischemia, and/or inflammation most commonly promotes angiogenesis but can also contribute to EndMT [29, 30]. 

*EndMT Inhibitors*. While most BMPs promote EndMT, BMP7 appears to be a negative regulator of EndMT [31], although the distinct differences between the downstream signals of the individual BMP isoforms remain elusive. Vascular Endothelial Growth Factor-A (VEGF-A) is known to inhibit EndMT through VEGFR2 signaling [32]. Inversely, VEGR1 can have a positive effect on EndMT by sequestering VEGF-A and preventing its interaction with VEGFR2 [33]. Recent evidence has shown that BMP signaling can also repress VEGF-A to help promote EndMT [34]. Fibroblast Growth Factor Receptor 1 (FGFR1) signaling can inhibit TGF-*β*-induced EndMT [35]. FGF-2, although found to be an inducer of EndMT in some types of endothelial cells [36], has also been shown to inhibit EndMT in others through miR-20a-mediated inhibition of TGF-*β* signaling [37].

MicroRNAs miR-15a, miR-23b, and miR-199a impair EndMT during heart development, although the miR-15a-dependent inhibition is only partial [38]. miR-126 blocks TGF-*β*1-induced EndMT of bone-marrow derived endothelial progenitor cells through direct targeting of the PI3K subunit p85 [39]. miR-155 impairs TGF-*β*-induced EndMT by inhibiting RhoA expression [40]. miR-302c negatively regulates expression of metadherin (MTDH) to impair EndMT associated with hepatocellular carcinoma [41]. N-acetyl-seryl-aspartyl-lysyl-proline (AcSDKP), a peptide substrate of angiotensin-converting enzyme (ACE), inhibits EndMT through the upregulation of microRNA let-7 and restoration of the FGF receptor [42].

Hydrogen sulfide can ameliorate EndMT caused by endoplasmic reticulum stress by activating the Src signaling pathway [43]. Aqueous extracts of* Psoralea corylifolia* L. have been shown to inhibit lipopolysaccharide-induced EndMT by inhibiting NF-*κ*B-dependent expression of Snail [44]. Glucagon-like peptide-1 (GLP-1) blocks high glucose-induced EndMT by reducing expression of reactive oxygen species (ROS) and inhibiting poly(ADP-ribose) polymerase 1 (PARP-1) [45]. The extracellular matrix protein fibulin-1 can suppress EndMT by reducing expression TGF-*β*2 [46]. High-density lipoproteins (HDL) have been shown to inhibit EndMT induced by TGF-*β*1 signaling [47].

Several drugs have been proposed as EndMT inhibitors. Linagliptin, a DPP-4 inhibitor that impairs its interaction with integrin *β*1, has been shown to block TGF-*β*2-induced EndMT [48]. Rapamycin blocks EndMT by suppressing the mTOR signaling pathway [49]. Relaxin (RLX) has been shown to inhibit isoproterenol-induced EndMT in a cardiac fibrosis model in rats through notch-mediated signaling [50]. Macitentan, an endothelin-1 receptor inhibitor, was shown to impair EndMT induced by either endothelin-1 or TGF-*β*1 [51]. Marimastat, a broad-spectrum MMP inhibitor, prevents FGF-2-dependent EndMT of corneal endothelial cells [52]. Kallistatin blocks TGF-*β*-induced EndMT through upregulation of endothelial nitric oxide synthase (eNOS) and by differential regulation of miR-21 [53]. Spironolactone, an aldosterone receptor blocker, can also inhibit TGF-*β*-induced EndMT by controlling Notch1 expression [54]. Scutellarin can also regulate Notch1 and Jagged1 expression to prevent isoprenaline-induced EndMT [55]. Losartan, an inhibitor of angiotensin II type 1 receptor, impairs EndMT by blocking TGF-*β* signaling [56]. Cinacalcet attenuates EndMT in cardiac fibrosis associated with elevated serum levels of parathyroid hormone (PTH) by suppressing the hormone levels [57]. Interestingly, hydrocortisone has been proposed to reverse EndMT through mesenchymal-endothelial transition by enhancing endothelial cell adhesion [58]. These functional inhibitors may be used as potential therapeutic agents to perturb the pathological effects of EndMT. 

*EndMT in Development and Disease*. EndMT has been shown to regulate angiogenesis [59], as well as cardiac development [60]. EndMT causes formation of the valves and septa of the heart during embryogenesis [60, 61]. In the postnatal organism, tissue damage and/or inflammation can stimulate this embryonic mechanism to give rise to fibroblasts and myofibroblasts that form scar tissue during wound healing or fibrotic diseases [2].

EndMT has a critical role in the generation of fibroblasts in kidney [62], lung [29], intestinal [63], and cardiac fibrosis [64]. This EndMT-dependent fibrotic phenotype contributes to diseases such as systemic sclerosis [65], atherosclerosis [66], pulmonary hypertension [67], diabetic nephropathy [68], diabetic retinopathy [69], sepsis [70], and cerebral cavernous malformations [71]. It also plays a central role in vein graft remodeling [72].

Further, while the epithelial-mesenchymal transition (EMT) has been shown to be the primary mechanism of cancer metastasis [73] and for the formation of cancer stem cells [74], EndMT occurs to form cancer-associated fibroblasts in the tumor microenvironment that help regulate the progression of the disease [75]. EndMT has also been proposed to have a role in the metastatic extravasation of cancer cells [76]. It may also have a part in central nervous system diseases associated with dysfunction of the blood-brain barrier [77]. 

*EndMT in the Generation of Connective Tissues*. Other than fibroblasts, recent studies have shown the ability of EndMT to generate various different types of connective tissues. Lineage tracing and biomarker studies have suggested an endothelial origin of heterotopic cartilage and bone that forms in a rare disease called fibrodysplasia ossificans progressiva (FOP) [5, 78, 79]. Patients with this disease carry a gain-of-function mutation in the gene encoding activin-like kinase 2 (ALK2) receptor [80]. Upon expressing this mutated gene in endothelial cells, they undergo EndMT and acquire properties of mesenchymal stem cells with the ability to transform into bone, cartilage, or fat cells [5]. A recent study has shown that kidney cells isolated from FOP patients can be transformed into induced pluripotent stem cells (iPSC) and subsequently differentiated into endothelial cells, which spontaneously underwent EndMT in culture [81].

The ability of EndMT to generate osteoprogenitor cells has also been observed in vascular calcifications [82, 83], valvular calcifications [84], and tumor calcifications [85]. Another recent study has shown that BMP6 has the ability to stimulate EndMT and subsequent differentiation to osteoblasts both independently and synergistically with oxidized low-density lipoprotein [86]. Tang et al. showed that high glucose levels mediate endothelial differentiation to chondrocytes through EndMT [87].

Lineage tracing studies using VE-cadherin-Cre reporter mice have demonstrated an endothelial origin of white and brown fat cells [88]. A recent study that isolated endothelium from vascular tumors showed that these cells spontaneously undergo EndMT in culture and have the ability to form adipocytes and mural cells such as pericytes and smooth muscle cells [89]. Endothelial progenitor cells (EPCs) have also been induced to undergo EndMT and transform into smooth muscle cells [90].

Endothelial plasticity has also been linked to generation of skeletal myocytes for muscle repair [91]. Furthermore, lineage tracing in Tie1-Cre and VE-cadherin-Cre reporter mice has demonstrated an endothelial origin of cardiomyocytes during cardiac homeostasis, which are proposed to arise by EndMT [92]. 

**Figure 1:**
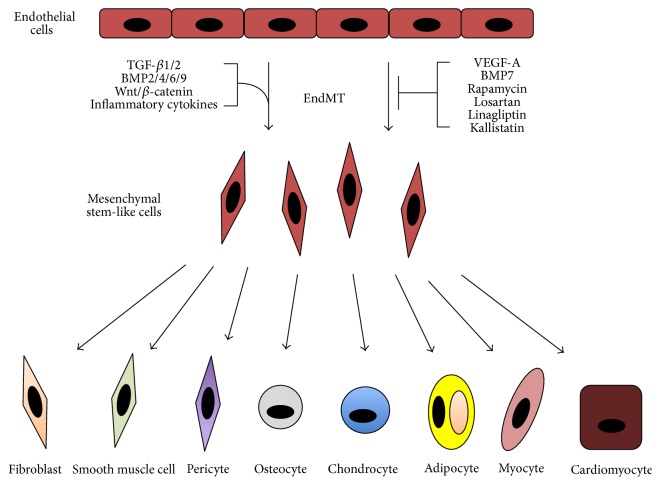
The multipotency of EndMT. Vascular endothelial cells are stimulated to undergo EndMT by various growth factors and inflammatory cytokines such as TGF-*β*s, BMPs, and Wnt. Proteins such as VEGF-A and BMP7, as well as drugs such as rapamycin, losartan, linagliptin, and kallistatin, can inhibit this cellular transformation. Endothelial-derived mesenchymal cells take on the properties of multipotent stem cells and can differentiate into fibroblasts, pericytes, smooth muscle, skeletal muscle, cardiac muscle, bone, cartilage, and fat cells.

*EndMT for Tissue Engineering and Regeneration*. The ability of EndMT to generate various different types of connective tissue (Figure 1) provides hope for using it as a potential method for tissue regeneration. For example, EndMT-dependent osteogenesis could be used to treat disorders such as osteoporosis or osteonecrosis. EndMT-induced chondrogenesis could be utilized for the treatment of osteoarthritis or temporal mandibular joint disorder (TMJD). Using EndMT to induce myogenesis could prove beneficial for muscular dystrophy, while cardiomyogenesis might be helpful for regenerating heart muscle after myocardial infarction. The process may also aid in vascular tissue regeneration, particularly in vasculogenesis through its ability to generate smooth muscle cells and pericytes. EndMT has already been found to be important in engineering cardiovascular tissue grafts through its ability to increase the production and remodeling of the extracellular matrix [93].

Tissue engineering* ex vivo* may be achieved through EndMT for the replacement of degenerated tissues. For personalized medicine, to avoid any potential host rejection, vascular endothelial cells can be easily obtained from patients from a skin sample. The tissue can be enzymatically digested and endothelial cells can be isolated using magnetic beads conjugated with endothelial-specific antibodies. These isolated endothelial cells can then be grown and expanded in culture and then loaded onto three-dimensional scaffolds composed of collagen, polylactic acid, hydrogel, and so forth. The endothelial cells can then be induced to undergo EndMT using any of the known cytokines that stimulate the transformation, followed by addition of differentiation medium to change the newly formed mesenchymal cells into the desired tissue type [94]. The engineered tissue may then be surgically transplanted into the patient.

For tissue regeneration* in vivo*, the potential use of EndMT is virtually endless since almost every tissue in the body is highly vascularized, so an abundant source of vascular endothelial cells should be present in damaged or degenerated tissues in need of repair. Drugs can be developed and locally applied to degenerated tissue to convert the vascular endothelium into the cell type of need. If some capillary blood vessels are lost during this cellular transformation, they should be naturally replenished through hypoxia-induced angiogenesis [95]. Therefore, EndMT should provide a natural and effective method for building new connective tissues from blood vessels.

## 2. Discussion

Although EndMT has positive effects in embryonic development and wound healing, it has traditionally been considered to have negative effects in disease. While most therapeutic studies attempt to inhibit the harmful effects of EndMT in progressive diseases such as cancer and fibrosis, it is now proposed that researchers harness this natural mechanism by inducing it for tissue regeneration for treatment of degenerative diseases. Although there may be potential risks of converting the vascular endothelium into other cell types for tissue regeneration, such as blood vessel leakage or cell death associated with hypoxia, the target tissue would already be degenerated and the natural mechanism of angiogenesis should replenish the blood vessels. Therefore, the potential benefits of restoring degenerated tissue using EndMT far outweigh the risks for regenerative medicine.
